# Association of Specific Leg Muscle Strength and Motor Features in Parkinson's Disease

**DOI:** 10.1155/2024/5580870

**Published:** 2024-06-20

**Authors:** Chatkaew Pongmala, Chernkhuan Stonsaovapak, Austin Luker, Alexis Griggs, Miriam van Emde Boas, Jacob M. Haus, Nicolaas I. Bohnen

**Affiliations:** ^1^Functional Neuroimaging, Cognitive and Mobility Laboratory, University of Michigan, Ann Arbor, MI, USA; ^2^Department of Radiology, University of Michigan, Ann Arbor, MI, USA; ^3^Morris K. Udall Center of Excellence in Parkinson's Disease Research, University of Michigan, Ann Arbor, MI 48105, USA; ^4^Department of Rehabilitation Medicine, Faculty of Medicine, Chulalongkorn University, Bangkok, Thailand; ^5^School of Kinesiology, University of Michigan, Ann Arbor, MI, USA; ^6^Department of Neurology, University of Michigan, Ann Arbor, MI, USA

## Abstract

**Background:**

Postural instability and gait difficulties (PIGD) are a significant cause of falls, mobility loss, and lower quality of life in Parkinson's disease (PD). The connection between PD progression and diminished strength in the lower limbs has been acknowledged. However, the identification of specific muscle groups linked to PIGD and non-PIGD motor features is still unknown.

**Objective:**

To explore the relationship between the strength of specific lower limb muscle groups, along with muscle mass, and their associations with PIGD, PIGD subtypes, and non-PIGD motor features in PD.

**Methods:**

95 PD participants underwent detailed motor and non-motor test batteries, including lower limb isometric strength testing and whole-body lean mass assessments. Correlation analysis and univariate and multivariate linear/logistic forward stepwise regression were performed to test associations between PIGD and non-PIGD motor features with normalized value (z-score) of lower limb muscle strength and measures of lean mass.

**Results:**

Multivariate regression analysis, adjusted for age, gender, and levodopa equivalent dose, revealed that hip abductor strength was significantly associated with overall PIGD motor severity ratings (*p* < 0.001), impaired balance (*p* < 0.001), and non-PIGD Parkinsonian motor features (*p* < 0.001). Conversely, hip extensor strength was significantly associated with falls, slow walking, and FoG motor features (*p*=0.016; *p*=0.003; *p*=0.020, respectively).

**Conclusion:**

We found that lower hip abductor strength was associated with PIGD and non-PIGD motor features. The association between non-PIGD motor features may suggest specific vulnerability of the hip abductors as part of a proposed brain-muscle loop hypothesis in PD. Moreover, lower hip extensor strength correlated with falls, slow walking, and FoG.

## 1. Introduction

Parkinson's disease (PD) is characterized by motor and non-motor symptoms which progress over time, causing increasing disability. The primary motor symptoms include Postural Instability and Gait Difficulties (PIGD), which can contribute to falls, loss of independence, and reduced quality of life. PIGD can be divided into four motor features: impaired balance, slow walking, falls, and freezing of gait (FoG) [1]. Although various disease-specific neurochemical factors have been identified, neuromuscular factors are also thought to play a role in contributing to PIGD in PD [2].

In older adults, decreased lower body strength is a predictor of gait and balance difficulties, including an increased likelihood of falls [3]. Sarcopenia, a condition characterized by decreased muscle strength, muscle mass, and physical function, is more prevalent in PD compared to an elderly control group [2]. A recent review of muscle strength studies in PD [4] found that PD stages and more severe motor symptoms were correlated with weaker lower limb muscles, reduced muscle strength, and reduced power compared to healthy controls. Additionally, fall and fracture risks are increased for people with PD partially due to decreased muscle strength in the lower limb muscles, with approximately 70% of PD patients falling annually [5]. Conversely, exercise has been shown to improve motor symptoms of PD by improving muscle strength and contributing to better balance and gait functions [6].

In locomotion, certain muscle groups serve as primary movers, like the knee extensor and ankle plantar flexor, while muscles around the hip joints such as the hip abductor are recognized as stabilizers [7]. Although general reduction in lower body strength has been correlated with stages of PD and increased fall risk, it remains unclear which movements or specific muscle groups of the lower body are the most impacted when the disease progresses. Understanding the links between muscle strength, lean mass, and motor impairments is a key component in improving PD-specific rehabilitation methods. To investigate these connections, our study aims to determine the association between the strength and lean mass of specific muscles in the lower extremity and the four main subtypes of PIGD motor features: (1) impaired balance, (2) falls, (3) slow walking, and (4) FoG, as well as non-PIGD Parkinsonian motor severity ratings. We hypothesize that hip muscle strength and lean muscular mass will exhibit the strongest association with PIGD motor features and non-PIGD motor features in patients with PD.

## 2. Methods

This study was conducted as a cross-sectional investigation. Idiopathic PD patients diagnosed by movement disorders neurologists were recruited from the University of Michigan and VA Ann Arbor Health System. The study protocol was reviewed and approved by the university's institutional review boards. Written informed consent was obtained from all patients.

The modified Hoehn and Yahr stages (H&Y), a simple staging assessment ranging from 1 to 5 used for the staging of the functional disability associated with PD, and the International Parkinson and Movement Disorder Society-Unified Parkinson's Disease Rating Scale II and III (MDS-UPDRS) were assessed during OFF state. Demographical data and clinical assessments such as levodopa equivalent dose (LED), disease duration, and history of falls were obtained during the visit.

Lower leg muscle strength was assessed by MicroFET2™ MMT. Hip flexor (iliopsoas), extensor (gluteus maximus), abductor (gluteus medius), and adductor, knee flexor (hamstrings) and extensor (quadriceps), and ankle plantar flexor (gastrocnemius) and dorsiflexor (tibialis anterior) were assessed each over three 5s trials. The maximum force value from the best trial for each muscle group was selected. Dual-energy X-ray absorptiometry (DXA) (Hologic Inc., Bedford, MA, USA) was used for the measurements of total lean mass, trunk lean mass, leg lean mass, appendicular lean mass (ALM), and appendicular lean mass index (ALMI).

### 2.1. Statistical Analysis

We performed a cross-sectional analysis to examine the association between the specific lower extremity muscle strength, lean mass, and the four main PIGD motor features: impaired balance, slow walking, falls, and FoG (all as dichotomous variables).

PIGD motor features were defined as the total score of MDS-UPDRS III items 3.10–3.12, which encompass the domains of gait, postural instability, and FoG. Non-PIGD motor features were defined as the total score of MDS-UPDRS III, excluding the scores for these PIGD items. Impaired balance was defined as Hoehn and Yahr (H&Y) ≥ 2.5 as previously reported [3]. Slow walking status was defined as a walking speed of less than 1 m/s. Fall status was determined by the history of falls in the preceding one year. FoG status was defined as MDS-UPDRS item 3.11 > 0 [1].

The normality of muscle strength and muscle mass was assessed using the Shapiro–Wilk test, complemented by histograms. Normalized values (Z-score) within this cohort study were calculated and obtained from SPSS and used for the mean and the standard deviation of lower extremity muscle strength and the measurements of total lean mass (kg), trunk lean mass (kg), leg lean mass (kg), appendicular lean mass (ALM), and appendicular lean mass index (ALMI). We employed Z-score normalization in our analysis to mitigate the effect of gender, which is recognized for its variance in muscle strength and mass between males and females [8]. Correlation analysis was conducted between the normalized strength (Z-score) of lower extremity muscles, ALM, ALMI, total lean mass, trunk lean mass, leg lean mass, PIGD motor features, and non-PIGD motor features. Pearson correlation was utilized to correlate two continuous variables, while the Point-Biserial correlation coefficient was employed to correlate continuous data with dichotomous data (four subtypes of PIGD motor features). We used Bonferroni correction for multiple comparisons. Univariate and multivariate regression analyses were performed to find the association between PIGD motor features, their subtypes (falls, slow walking, imbalance, and FoG), and non-PIGD motor features as dependent variable and normalized strength (*Z*-score) of specific lower extremity muscles and lean mass from DXA. Variables with *p* < 0.2 at the univariate regression analysis were recruited to perform multivariate forward stepwise regression models. All multivariate regression models were adjusted for age and gender and LED. Multicollinearity analysis was used to find the correlation between variables before performing multivariate regression analysis.

All tests were two-tailed with a *p* value <0.05. Statistical analyses were performed using SPSS (version 29) statistical software.

## 3. Results

95 participants (74 males and 21 females) with PD (mean age 67.41 ± 6.13 years, mean disease duration at 7.57 ± 4.93 years, and mean LED of 714.16 ± 410.13 mg) were included in the study. Demographic, muscle strength, lean mass, and clinical details of PD patients are shown in Table 1. In addition, we also found that normalized values (*Z*-score) of ALM, ALMI, total lean mass, trunk lean mass, and leg lean mass were positively correlated with normalized strength (*Z*-score) of all lower extremity muscles (*p* < 0.05) (Table 2). After correction for multiple testing with the Bonferroni method, hip extensors, ankle dorsiflexors, and ankle plantar flexors were not found to be significantly correlated with any of the muscle mass variables.

### 3.1. PIGD Motor Features

In correlation analysis, normalized strength of hip abductor (*r* = −0.347, *p*=0.001) and knee extensor (*r* = −0.205, *p*=0.049) was found to be negatively correlated with PIGD motor features. No lean body mass was correlated with PIGD motor features. See Table 3 for more details. However, after adjusting for multiple correlation by using Bonferroni corrections, only normalized strength of hip abductor was found to be negatively correlated with PIGD motor features.

In regression analysis, normalized strength of hip extensor (*β* = −0.313, *p*=0.125), hip abductor (*β* = −0.552, *p*=0.006), hip adductor (*β* = −0.450, *p*=0.024), and knee flexor (*β* = −0.273, *p*=0.179), ALMI (*β* = −0.307, *p*=0.132), and leg lean mass (*β* = −0.287, *p*=0.160), identified through univariate linear regression analysis, were included in a multivariate linear forward stepwise regression. Multivariate linear forward stepwise regression analysis after adjusting for age and LED demonstrated that normalized strength of hip abductor (*β* = −0.686, *p* < 0.001) was significantly associated with PIGD motor features. See Table 4 for more details.

### 3.2. PIGD Motor Features: Impaired Balance

In correlation analysis, normalized strength of hip extensor (*r* = −0.255, *p*=0.015), hip abductor (*r* = −0.379, *p* < 0.001), hip adductor (*r* = −0.336, *p*=0.001), knee flexor (*r* = −0.286, *p*=0.005), and knee extensor (*r* = −0.289, *p*=0.005) was found to be negatively correlated with impaired balance significantly. See Table 3 for more details. In addition, ALM (*r* = −0.232, *p*=0.025), ALMI (*r* = −0.302, *p*=0.003), total lean mass (*r* = −0.272, *p*=0.008), and leg lean mass (*r* = −0.297, *p*=0.004) were also negatively correlated with impaired balance significantly (Table 3). However, after adjusting for multiple correlation by using Bonferroni corrections, normalized strength of hip abductor and hip adductor and ALMI were found to be negatively correlated with impaired balance.

In regression analysis, normalized strength of hip flexor (OR = 0.701, *p*=0.107), hip extensor (OR = 0.574, *p*=0.019), hip abductor (OR = 0.417, *p*=0.001), hip adductor (OR = 0.469, *p*=0.002), knee flexor (OR = 0.535, *p*=0.008), knee extensor (OR = 0.532, *p*=0.007), and ankle dorsiflexor (OR = 0.653, *p*=0.055), ALM (OR = 0.604, *p*=0.029), ALMI (OR = 0.504, *p*=0.005), total lean mass (OR = 0.543, *p*=0.011), trunk lean mass (OR = 0.660, *p*=0.066), and leg lean mass (OR = 0.509, *p*=0.006), identified through univariate logistic regression analysis, were included in a multivariate logistic forward stepwise regression. Multivariate logistic forward stepwise regression analysis after adjusted for age and LED demonstrated that normalized strength of hip abductor (OR = 0.360, *p* < 0.001) was significantly associated with impaired balance. See Table 5 for more details.

### 3.3. PIGD Motor Features: Falls

In correlation analysis, normalized strength of hip flexor (*r* = −0.226, *p*=0.03), hip extensor (*r* = −0.275, *p*=0.01), hip abductor (*r* = −0.222, *p*=0.035), and knee extensor (*r* = −0.252, *p*=0.016) was found to be negatively correlated with falls significantly. See Table 3 for more details. No lean body mass measures were correlated with falls (Table 3). However, after adjusting for multiple correlation by using Bonferroni corrections, no variable was found to be negatively correlated with falls.

In regression analysis, normalized strength of hip flexor (OR = 0.622, *p*=0.035), hip extensor (OR = 0.548, *p*=0.012), hip abductor (OR = 0.620, *p*=0.040), knee flexor (OR = 0.622, *p*=0.035), knee extensor (OR = 0.583, *p*=0.02), ankle plantar flexor (OR = 0.700, *p*=0.107), and ankle dorsiflexor (OR = 0.700, *p*=0.104), identified through univariate logistic regression analysis, was included in a multivariate logistic forward stepwise regression. No variables from lean mass yielded *p* values below 0.2. Multivariate logistic forward stepwise regression analysis after adjusting for age and LED demonstrated that normalized strength of hip extensor (OR = 0.562, *p*=0.016) was significantly associated with falls. See Table 5 for more details.

### 3.4. PIGD Motor Features: Slow Walking

In correlation analysis, normalized strength of hip extensor (*r* = −0.287, *p*=0.006), hip abductor (*r* = −0.297, *p*=0.004), hip adductor (*r* = −0.261, *p*=0.011), and knee flexor (*r* = −0.244, *p*=0.018) was found to be negatively correlated with slow walking significantly. See Table 3 for more details. No lean body mass measures were correlated with slow walking (Table 3). However, after adjusting for multiple correlation by using Bonferroni corrections, no variable was found to be negatively correlated with slow walking.

In regression analysis, normalized strength of hip flexor (OR = 0.689, *p*=0.128), hip extensor (OR = 0.484, *p*=0.009), hip abductor (OR = 0.447, *p*=0.006), hip adductor (OR = 0.507, *p*=0.015), knee flexor (OR = 0.541, *p*=0.022), knee extensor (OR = 0.658, *p*=0.1), ankle plantar flexor (OR = 0.665, *p*=0.109), and ankle dorsiflexor (OR = 0.652, *p*=0.091) and leg lean mass (OR = 0.727, *p*=0.173), identified through univariate logistic regression analysis, were included in a multivariate logistic forward stepwise regression. Multivariate logistic forward stepwise regression analysis after adjusting for age and LED demonstrated that normalized strength of hip extensor (OR = 0.382, *p*=0.003) was significantly associated with slow walking. See Table 5 for more details.

### 3.5. PIGD Motor Features: Freezing of Gait

In correlation analysis, no correlations were found between normalized strength of any leg muscles, body lean mass measures, and freezing of gait. See Table 3 for more details.

In regression analysis, normalized strength of hip extensor (OR = 0.627, *p*=0.168), identified through univariate logistic regression analysis, was included in a multivariate logistic forward stepwise regression. No body lean mass measures yielded *p* values below 0.2. Multivariate logistic forward stepwise regression analysis after adjusting for age and LED demonstrated that normalized strength of hip extensor (OR = 0.364, *p*=0.02) was significantly associated with freezing of gait. See Table 5 for more details.

### 3.6. Non-PIGD Motor Features

In correlation analysis, normalized strength of hip abductor (*r* = −0.286, *p*=0.006) and hip adductor (*r* = −0.234, *p*=0.024) was found to be negatively correlated with non-PIGD motor symptoms. No lean body mass measures were correlated with non-PIGD motor features. See Table 3 for more details. However, after adjusting for multiple correlation by using Bonferroni corrections, no variable was found to be negatively correlated with non-PIGD motor features.

In regression analysis, normalized strength of hip extensor (*β* = −1.638, *p*=0.189), hip abductor (*β* = −4.059, *p*=0.001), hip adductor (*β* = −2.280, *p*=0.061), knee flexor (*β* = −1.746, *p*=0.153), knee extensor (*β* = −2.316, *p*=0.049), and ankle dorsiflexor (*β* = −1.561, *p*=0.199) was identified through univariate linear regression analysis and included in a multivariate linear forward stepwise regression. Notably, no variables from lean mass yielded *p* values below 0.2. Multivariate linear forward stepwise regression analysis after adjusting for age and LED demonstrated that normalized strength of hip abductor (*β* = −4.790, *p* < 0.001) was significantly associated with non-PIGD motor features. See Table 4 for more details.

## 4. Discussion

This is the first study to examine the association between sarcopenia, low muscle strength, lean mass, and their impact on PIGD and non-PIGD motor features in patients with PD. In this study, we identified an association between hip abductor strength and PIGD, non-PIGD, and impaired balance motor features. Furthermore, there was a tendency for muscle mass to be associated with balance impairment. Meanwhile, hip extensor muscle strength was found to be linked with other subtypes of PIGD motor features: falls, slow walking, and FoG.

The results indicated that there was a tendency for muscle strength in the proximal parts of the lower limb to have a negative correlation with worsening of motor symptoms which corresponds with a previous study [4]. However, among these factors, only the strength of the hip abductor muscle was identified as a significant independent factor strongly associated with overall PIGD motor features and specifically with balance impairments as the disease advanced. The hip abductor muscle is crucial for maintaining lateral balance stability and is often referred to as a stabilizer muscle due to its role in stabilizing the hip joint. It functions by stabilizing the torso over the standing leg and assisting in the restoration of dynamic balance through stepping reactions in the mediolateral plane, responding to perturbations [9, 10]. There is evidence indicating that the strength of the hip abductor and adductor muscles, which serve as stabilizers, experiences a more pronounced decline with age compared to other primary muscles [7]. However, our study reveals that hip abductor strength is an age-independent factor associated with motor symptoms in PD.

In a previous study, a moderate correlation was found between hip abductor muscle power and fast walking and choice stepping, indicating the muscle's role in pelvic stabilization and maintaining balance within the base of support [11]. The modified H&Y motor severity staging reflects the extent of a patient's affected side and impaired postural reflexes, with more advanced disease stages indicating increased involvement in axial motor function and postural reflexes. Our findings indicated a correlation between impaired postural reflexes and decreased strength of the hip abductor muscle, which plays a crucial role in maintaining pelvic stability within the base of support. Not only were PIGD motor features found to be correlated with hip abductor muscle strength, but also non-PIGD motor features. This finding was unexpected and may suggest specific vulnerability of the hip abductors as part of the proposed brain-muscle loop hypothesis. More recently, a neural network imaging study has identified the so-called [12] somato-cognitive action network alternating with effector regions in the motor cortex that may be in part related to the existence of such brain-muscle loop in PD [13].

Another crucial discovery in this study is the link between diminished hip extensor muscle strength and other PIGD motor features including falls, slow walking, and FoG. The hip extensor muscle plays a vital role in dynamic stability and everyday functional activities like standing up, walking, and maintaining an upright posture. During normal gait, the hip extensor muscle engages in the initial phase of the stance, inducing a posterior pelvic tilt. Decreased strength in this muscle among individuals with PD might contribute to an exaggerated anterior pelvic tilt and forward trunk flexion [14]. Such abnormal posture could lead to instability in balance and increase the risk of falls. A previous study indicated that individuals with PD exhibited weaker hip extensor muscle strength than knee extensor muscle strength compared to the control group, supporting the notion of a more significant impairment in axial muscles rather than distal motor function in PD [15]. As a result, PD patients encounter difficulties in standing up from chairs due to lower limb weakness, particularly in the hip extensor muscle, potentially heightening the risk of falls [15, 16]. Furthermore, the hip extensor emerged as the primary differentiator in assessing balance recovery performance when compared to other lower limb muscles [17]. Taking these perspectives into account, early identification of declining strength in the hip extensor muscle could serve as a valuable indicator for impairment of dynamic balance stability and be associated with the risk of falls in individuals with PD.

We also observed that the decline in hip extensor muscle strength associated with slow walking speed in PD corresponds to previous findings in elderly men, where hip extensor muscle torque was a significant independent predictor for free walking velocity, stride length, and cadence [18]. In this study, we found that this association was independent of age in patients with PD. Additionally, decreased hip muscle strength was associated with FoG, which differs from slow gait speed in that the reduction in propulsive movement is much greater. Freezers tend to exhibit a flexed walking pattern characterized by hip and knee flexion, ankle dorsiflexion, and decreased stride length, which may be associated with weakness of the hip extensor muscle. While patients with PD perceive freezing as an inability to actively initiate the swing phase, the absence of push-off and increased flexion in the lower limbs are components of FoG [19].

Advanced PD often leads to sedentary behavior, potentially resulting in disused muscle atrophy. Antigravity muscles like the hip extensor, knee extensor, and ankle plantar flexor are expected to be affected earlier than other muscles [20], but this does not totally align with our findings: we found that hip abductor and hip extensor muscles were the worst when the disease progressed. The causes of decreasing muscle power in PD are still under scrutiny. However, some evidence supports the notion that it results from reduced muscle strength and bradykinesia induced by neurochemical changes in the brain [11]. There is also evidence supporting the link between brain pathology and muscle dysfunction in frail elderly individuals [12]. Conversely, an alternative perspective from our results suggests that the loss of strength in the hip abductor and hip extensor muscles might elucidate the clinical manifestation of PIGD motor features, particularly noticeable in the advanced stages of PD. The recent systematic review revealed that only a limited number of studies investigated the relationship between muscle strength and the severity of motor impairments [4]. Moreover, unlike our study, not all lower limb muscles were examined in those studies.

Sarcopenia is defined as the loss of both muscle mass and strength [2]. In a sarcopenic state, reductions in strength, indicative of muscle function, are reflective of decreased mass. Our findings indicate a significantly positive correlation between specific muscle strength of the lower limbs and ALMI, as well as total, trunk, and leg lean mass. Although there was a tendency for an association between lean mass and subtype impaired balance in PIGD motor features, it did not play a significant role as the same as muscle strength. Our findings are consistent with a 3-year prospective study on aging by Goodpaster [21], indicating that while the reduction in muscle mass is linked to diminishing strength in older adults, the decline in strength occurs at a much faster rate than the simultaneous loss of muscle mass, signifying a deterioration in muscle quality. As PD progresses, muscle quality tends to decrease before a reduction in muscle mass becomes apparent through body composition examination, with strength proving to be a more practical test than DXA. Therefore, objective muscle strength testing should be included in real practice for follow-up and early detection of sarcopenia conditions.

### 4.1. Significant Outcomes

We have established a correlation between reduced hip abductor and hip extensor strength and PIGD motor features. Consequently, targeted assessment and training should prioritize these muscle groups, particularly in the early stages of PD. The goal is to maintain or improve mediolateral stability, reduce the risk of falls, and enhance gait pattern and walking speed. Clinicians should be vigilant in evaluating the strength of these specific muscle groups rather than focusing solely on muscle lean mass in PD for early detection and tailored intervention. However, we recognize that gait and balance are complex processes that rely on much more than isolated muscle groups. Recommendations from this study for PD rehabilitation strategies would add particular focus to hip muscle strength in conjunction with more general physical therapy programs already in practice for PD patients.

### 4.2. Study Limitations

This cross-sectional study offers valuable insights into the correlation between various factors, yet its limitations warrant careful consideration. As a cross-sectional analysis, the study's ability to establish causality or infer a strong association might be constrained compared to prospective studies. Additionally, a notable limitation is the distribution of participants, with the majority falling within H&Y stages 2 and 3, with only 14% representing the early and late stages of PD. Moreover, there is a small number of women in this analysis. One approach we could take is to conduct a post hoc sensitivity analysis limited to males.

### 4.3. Future Recommendations

Conducting a longitudinal study is crucial to substantiate the link between declining muscle strength and disease progression. Investigating the root causes of muscle weakness is imperative, delving deeper into whether it stems from sedentary behavior, neurochemical changes in brain pathology, or a combination thereof. Such comprehensive exploration would greatly enhance our understanding of the intricate relationship between muscular decline and its underlying mechanisms within the context of disease progression. Furthermore, we recommend examining the relationship between muscle cross-sectional area or limb volume, along with muscle mass, and the progression of the disease.

## 5. Conclusion

Our findings revealed a link between reduced hip abductor strength and PIGD motor features, particularly with impaired balance, and non-PIGD motor features in patients with PD. The latter finding was unexpected and may suggest specific vulnerability of the hip abductors as part of a proposed brain-muscle loop hypothesis. Additionally, decreased hip extensor strength correlated with falls, slow walking, and FoG. Directing interventions toward strengthening these specific muscle groups could potentially prevent falls and improve gait and postural stability in individuals with PD.

## Figures and Tables

**Table 1:** Demographic and clinical details of PD patients, categorized by PIGD motor subtypes.

Parameters	All PD	Normal balance	Impaired balance	No falls	Falls	Normal walking	Slow walking	No FoG	FoG
	*n* = 95	*n* = 36	*n* = 59	*n* = 50	*n* = 42	*n* = 69	*n* = 26	*n* = 82	*n* = 13
	Mean ± SD	Mean ± SD	Mean ± SD	Mean ± SD	Mean ± SD	Mean ± SD	Mean ± SD	Mean ± SD	Mean ± SD
Age (yrs)	67.41 ± 6.13	66.42 ± 5.68	68.02 ± 6.35	67.60 ± 5.77	67.40 ± 6.75	67.26 ± 5.39	67.81 ± 7.87	67.20 ± 6.09	68.77 ± 6.41
Gender (M/F)	74/21	33/3	41/18	43/7	28/14	56/13	18/8	63/19	11/2
Disease duration (yrs)	7.57 ± 4.93	7.43 ± 5.36	7.66 ± 4.70	5.96 ± 3.49	9.27 ± 5.58	7.31 ± 4.77	8.27 ± 5.37	7.01 ± 4.60	11.15 ± 5.61
Modified H&Y	2.5	2	2.5	2	2.5	2.5	2.75	2.5	3
LED (mg)	714.16 ± 410.13	632.20 ± 391.73	764.17 ± 416.29	678.68 ± 408.74	741.93 ± 357.33	660.00 ± 391.01	857.88 ± 432.47	638.72 ± 348.65	1,190.00 ± 461.47
Hip flexor strength (*N*)	198.41 ± 55.63	210.30 ± 43.98	191.15 ± 60.89	210.18 ± 52.24	184.73 ± 58.69	203.79 ± 53.59	184.14 ± 59.44	200.28 ± 54.89	186.62 ± 61.07
Hip extensor strength (*N*)	126.10 ± 46.33	141.56 ± 47.07	117.15 ± 43.84	136.52 ± 48.16	111.52 ± 39.10	134.31 ± 44.94	104.76 ± 43.78	128.76 ± 46.66	108.80 ± 41.77
Hip abductor strength (*N*)	117.43 ± 47.20	139.62 ± 47.90	103.16 ± 41.19	126.54 ± 49.76	105.68 ± 41.70	126.18 ± 46.78	95.21 ± 41.29	118.19 ± 47.01	112.35 ± 50.29
Hip adductor strength (*N*)	94.31 ± 37.47	110.06 ± 35.65	84.36 ± 35.39	94.96 ± 29.98	91.03 ± 43.64	100.37 ± 36.36	78.68 ± 36.39	96.16 ± 37.69	81.77 ± 34.83
Knee flexor strength (*N*)	118.72 ± 45.68	135.21 ± 40.05	108.48 ± 46.27	126.04 ± 46.04	108.23 ± 43.60	125.39 ± 44.44	100.28 ± 44.79	119.67 ± 45.34	112.78 ± 49.22
Knee extensor strength (*N*)	215.16 ± 75.61	242.79 ± 64.44	198.01 ± 77.43	232.84 ± 75.63	194.32 ± 73.47	223.19 ± 70.27	194.16 ± 86.02	215.94 ± 73.19	210.33 ± 92.52
Ankle plantar flexor strength (*N*)	206.49 ± 67.62	215.69 ± 63.71	200.78 ± 69.87	215.73 ± 67.08	192.51 ± 67.71	213.46 ± 65.42	188.26 ± 71.16	207.62 ± 66.74	199.45 ± 75.36
Ankle dorsiflexor strength (*N*)	134.88 ± 57.21	149.46 ± 52.94	125.82 ± 58.33	143.55 ± 57.54	123.54 ± 57.29	141.11 ± 55.77	118.58 ± 58.81	136.15 ± 56.84	126.95 ± 61.22
ALM (kg)	19.11 ± 2.62	19.89 ± 2.55	18.64 ± 2.57	19.11 ± 2.65	19.13 ± 2.71	19.14 ± 2.64	19.02 ± 2.64	19.09 ± 2.74	19.22 ± 1.81
ALMI (kg/m^2^)	8.34 ± 1.39	8.88 ± 1.35	8.02 ± 1.32	8.45 ± 1.38	8.25 ± 1.43	8.40 ± 1.44	8.18 ± 1.26	8.36 ± 1.45	8.23 ± 0.91
Total lean mass (kg)	58.69 ± 11.50	62.72 ± 10.09	56.29 ± 11.69	59.79 ± 10.82	57.34 ± 12.59	59.48 ± 11.57	56.60 ± 11.27	58.84 ± 11.98	57.73 ± 8.17
Trunk lean mass (kg)	28.91 ± 5.70	30.34 ± 5.12	28.07 ± 5.89	29.20 ± 5.46	28.45 ± 6.13	29.15 ± 5.62	28.30 ± 5.96	28.92 ± 5.88	28.87 ± 4.63
Leg lean mass (kg)	9.55 ± 2.05	10.34 ± 1.75	9.09 ± 2.08	9.80 ± 1.89	9.31 ± 2.25	9.73 ± 2.11	9.09 ± 1.82	9.60 ± 2.11	9.29 ± 1.64

PD: Parkinson's disease, H&Y: Hoehn and Yahr, LED: levodopa equivalent dose, MDS-UPDRS: International Parkinson and Movement Disorder Society-Unified Parkinson's Disease Rating Scale, HF: hip flexor, HE: hip extensor, HABD: hip abductor, HADD: hip adductor, KF: knee flexor, KE: knee extensor, APD: ankle plantar flexor, and ADF: ankle dorsiflexor.

**Table 2:** Correlations between normalized values of lower extremity muscle strength, appendicular lean mass (ALM), appendicular lean mass index (ALMI), total lean mass, trunk lean mass, and leg lean mass.

		ALM	ALMI	Total lean mass	Trunk lean mass	Leg lean mass
HF	*r*	0.437^*∗∗*^	0.446^*∗∗*^	0.512^*∗∗*^	0.501^*∗∗*^	0.496^*∗∗*^
	*p* value	**<0.001**	**<0.001**	**<0.001**	**<0.001**	**<0.001**

HE	*r*	0.261^*∗*^	0.255^*∗*^	0.259^*∗*^	0.246^*∗*^	0.242^*∗*^
	*p* value	0.014	0.016	0.014	0.020	0.022

HABD	*r*	0.303^*∗∗*^	0.296^*∗∗*^	0.323^*∗∗*^	0.322^*∗∗*^	0.281^*∗∗*^
	*p* value	0.004	0.004	**0.002**	**0.002**	0.007

HADD	*r*	0.367^*∗∗*^	0.358^*∗∗*^	0.316^*∗∗*^	0.292^*∗∗*^	0.307^*∗∗*^
	*p* value	**<0.001**	**<0.001**	**0.002**	0.005	**0.003**

KF	*r*	0.418^*∗∗*^	0.405^*∗∗*^	0.418^*∗∗*^	0.415^*∗∗*^	0.361^*∗∗*^
	*p* value	**<0.001**	**<0.001**	**<0.001**	**<0.001**	**<0.001**

KE	*r*	0.350^*∗∗*^	0.346^*∗∗*^	0.379^*∗∗*^	0.382^*∗∗*^	0.349^*∗∗*^
	*p* value	**0.001**	**0.001**	**<0.001**	**<0.001**	**0.001**

APF	*r*	0.272^*∗∗*^	0.276^*∗∗*^	0.267^*∗∗*^	0.253^*∗*^	0.233^*∗*^
	*p* value	0.008	0.007	0.010	0.015	0.025

ADF	*r*	0.238^*∗*^	0.215^*∗*^	0.249^*∗*^	0.257^*∗*^	0.204^*∗*^
	*p* value	0.022	0.038	0.016	0.013	0.050

^*∗*^ *p* < 0.05; ^*∗∗*^*p* < 0.01. HF: hip flexor, HE: hip extensor, HABD: hip abductor, HADD: hip adductor, KF: knee flexor, KE: knee extensor, APF: ankle plantar flexor, ADF: ankle dorsiflexor, ALM: appendicular lean mass, and ALMI: appendicular lean mass index. Significant variables after Bonferroni correction are shown in bold font.

**Table 3:** Correlations between PIGD, PIGD subtypes, non-PIGD motor features, and normalized values of lower extremity muscle strength, appendicular lean mass (ALM), appendicular lean mass index (ALMI), total lean mass, trunk lean mass, and leg lean mass.

		HF	HE	HABD	HADD	KF	KE	APF	ADF	ALM	ALMI	Total lean mass	Trunk lean mass	Leg lean mass
PIGD	*r*	−0.024	−0.140	−0.347^*∗∗*^	−0.196	−0.149	−0.205^*∗*^	−0.063	−0.134	0.090	0.092	0.074	0.065	0.096
	*p* value	0.817	0.189	**0.001**	0.061	0.153	0.049	0.546	0.199	0.391	0.380	0.480	0.537	0.359

Imbalance	*r*	−0.168	−0.255^*∗*^	−0.379^*∗∗*^	−0.336^*∗∗*^	−0.286^*∗∗*^	−0.289^*∗∗*^	−0.108	−0.202	−0.232^*∗*^	−0.302^*∗∗*^	−0.272^*∗∗*^	−0.193	−0.297^*∗∗*^
	*p* value	0.104	0.015	**<0.001**	**0.001**	0.005	0.005	0.301	0.051	0.025	**0.003**	0.008	0.062	0.004

Fall	*r*	−0.226^*∗*^	−0.275^*∗∗*^	−0.222^*∗*^	−0.054	−0.195	−0.252^*∗*^	−0.171	−0.173	0.003	−0.071	−0.105	−0.065	−0.117
	*p* value	0.030	0.010	0.035	0.613	0.063	0.016	0.105	0.101	0.977	0.501	0.321	0.540	0.269

Slow walking	*r*	−0.158	−0.287^*∗∗*^	−0.297^*∗∗*^	−0.261^*∗*^	−0.244^*∗*^	−0.173	−0.168	−0.177	−0.021	−0.072	−0.113	−0.067	−0.142
	*p* value	0.125	0.006	0.004	0.011	0.018	0.096	0.106	0.088	0.843	0.488	0.280	0.522	0.173

FoG	*r*	−0.085	−0.147	−0.042	−0.129	−0.052	−0.026	−0.042	−0.056	0.018	−0.031	−0.033	−0.003	−0.053
	*p* value	0.414	0.166	0.692	0.216	0.616	0.806	0.688	0.593	0.865	0.764	0.750	0.979	0.614

No PIGD	*r*	−0.058	−0.163	−0.286^*∗∗*^	−0.234^*∗*^	−0.140	−0.084	−0.053	−0.038	−0.059	−0.157	−0.093	−0.013	−0.146
	*p* value	0.574	0.125	0.006	0.024	0.179	0.419	0.613	0.720	0.572	0.132	0.371	0.901	0.160

^*∗*^ *p* < 0.05; ^*∗∗*^*p* < 0.01. PIGD: postural instability and gait difficulties, FoG: freezing of gait, HF: hip flexor, HE: hip extensor, HABD: hip abductor, HADD: hip adductor, KF: knee flexor, KE: knee extensor, APF: ankle plantar flexor, ADF: ankle dorsiflexor, ALM: appendicular lean mass, and ALMI: appendicular lean mass index. Significant variables after Bonferroni correction are shown in bold font.

**Table 4:** Multivariate linear forward stepwise regression for PIGD and non-PIGD motor features adjusted for age and LED.

	Variables	Multivariate analysis				
		*β*	95% CI		*t*	*p* value
			Lower	Upper		
PIGD	HABD	−0.686	−1.055	−0.318	−3.704	<0.001
No PIGD	HABD	−4.790	−6.991	−2.589	−4.329	<0.001

LED: levodopa equivalent dose, PIGD: postural instability and gait difficulties, and HABD: hip abductor.

**Table 5:** Multivariate logistic forward stepwise regression for 4 PIGD motor subtypes adjusted for age and LED.

PIGD subtypes	Variables	Multivariate analysis				
		OR	95% CI		Wald	*p* value
			Lower	Upper		
Impaired balance	HABD	0.360	0.204	0.636	12.379	<0.001
Falls	HE	0.562	0.352	0.900	5.754	0.016
Slow walking	HE	0.380	0.203	0.713	9.083	0.003
FoG	HE	0.364	0.155	0.852	5.423	0.020

LED: levodopa equivalent dose, PIGD: postural instability and gait difficulties, FoG: freezing of gait, HABD: hip abductor, and HE: hip extensor.

## Data Availability

Data are available on request due to privacy/ethical restrictions.
